# Small Bowel Obstruction Caused by Enteroliths Formed in the Duodenal Diverticulum

**DOI:** 10.1002/jgh3.70125

**Published:** 2025-03-17

**Authors:** Jing Yu, Sheng‐Yi Zhu, Jing‐Jing Li, Lin‐Hua Yao

**Affiliations:** ^1^ Department of Gastroenterology, The First People's Hospital of Huzhou First Affiliated Hospital of Huzhou University Huzhou Zhejiang China

## Abstract

**Background and Objective:**

Small bowel obstruction is a common acute abdomen. The disease presentation changes rapidly and differential diagnosis is difficult. If diagnosis and treatment are delayed or inappropriate, the consequences will be serious.

**Methods:**

Here, we report here a rare case of small bowel obstruction caused by enteroliths formed in the duodenal diverticulum.

**Results:**

Conservative treatment was not effective. Six days later, the patient underwent emergency exploratory laparotomy to confirm small bowel obstruction, and enterotomy for lithotomy was performed.

**Conclusion:**

Obstruction of the small intestine due to passage of enteroliths from the duodenal diverticulum is rare. There is currently no definitive evidence delineating the optimal duration for non‐surgical treatment. However, it should be noted that the postponement of surgical intervention may elevate mortality rates.

## 1 Introduction

Small bowel obstruction is a common acute abdomen, with common symptoms of abdominal pain, vomiting, abdominal distension, anal exhaust, and cessation of defecation. The disease presentation changes rapidly, and differential diagnosis is difficult. If diagnosis and treatment are delayed or inappropriate, the consequences will be serious [1]. Here, we report here a rare case of small bowel obstruction caused by enteroliths formed in the duodenal diverticulum.

## 2 Case Report

**FIGURE 1:**
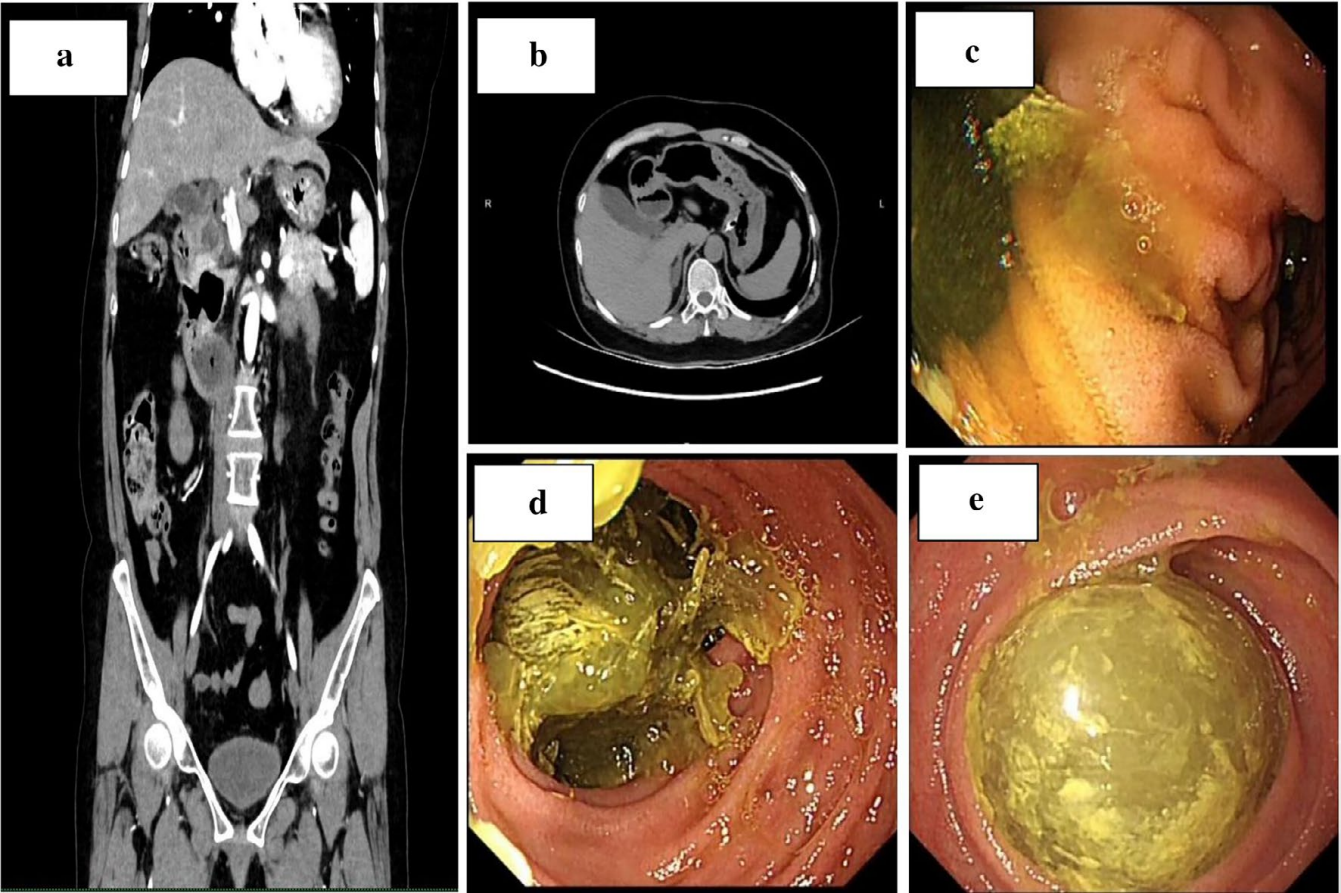
(a) Contrast‐enhanced computed tomography. Duodenal bulbar dilatation with diverticulum (coronal view). (b) Contrast‐enhanced computed tomography. Duodenal bulbar dilatation with diverticulum (axial view). (c) Gastroscopy. Duodenal diverticulum. (d) Gastroscopy. Duodenal obstruction. (e) Single‐balloon enteroscopy. A 2.5‐cm globular enterolith 280 cm from the pylorus.

**FIGURE 2:**
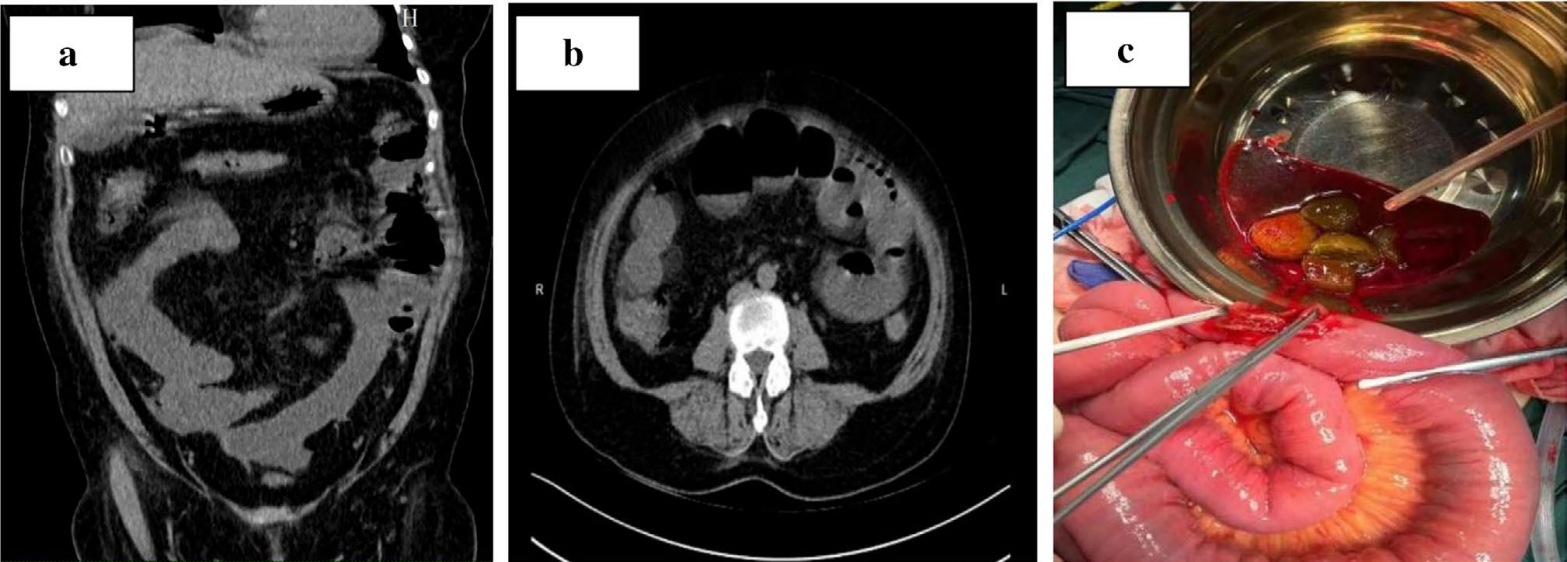
(a) Computed tomography. Small bowel obstruction with suspected ileocecal wall thickening (coronal view). (b) Computed tomography. Small bowel obstruction with suspected ileocecal wall thickening (axial view). (c) During the operation, exploratory laparotomy and small bowel incisions were performed to remove fecal stones.

A 55‐year‐old woman presented to the hospital with vomiting and diarrhea that had not resolved for 4 days. Ultrasonography at another hospital revealed a coarse wall of the gallbladder, a total white blood cell count of 21.1 × 10^9^/L, and a C‐reactive protein of 135.8 mg/L. Her past medical history was unremarkable, and physical examination showed no obvious positive signs. Contrast‐enhanced computed tomography (CECT) showed dilatation of the duodenal bulb with multiple diverticula and unclear visualization of the horizontal segment of the duodenum (Figure 1a,b). Gastroscopy revealed duodenal diverticulum (Figure 1c) with duodenal obstruction (Figure 1d). One day later, single‐balloon enteroscopy revealed a globular enterolith of approximately 2.5 cm located 280 cm from the pylorus (Figure 1e). The patient opted for conservative treatment with an indwelling nasogastric tube. Six days later, the patient did not exhaust or defecate, and physical examination showed abdominal distention, percussion drum sounds, and weak bowel sounds. Conservative treatment was not effective. Re‐examination of CT showed small bowel obstruction with suspected ileocecal wall thickening (Figure 2a,b). Within 24 h, the patient underwent emergency exploratory laparotomy to confirm small bowel obstruction, and enterotomy for lithotomy was performed (Figure 2c).

## 3 Discussion

For small bowel obstruction, the consensus among the Eastern American Association of Trauma Surgeons (EAST) and the World Society of Emergency Surgery (WSES) in the United States advocates for an initial trial of non‐surgical management in the absence of peritonitis, intestinal necrosis, or ischemia. Although there is currently no definitive evidence delineating the optimal duration for non‐surgical treatment, most experts concur that a period of 3–5 days is both safe and appropriate. It should be noted that postponing surgical intervention may elevate mortality rates [1]. Clinically, it is impossible to differentiate EI from gallstone ileus. Therefore, the absence of gallstones and the presence of a small intestinal diverticulum in the biliary tree need to be determined simultaneously to make a correct diagnosis [2]. In this patient, CT and gastroscopy had confirmed small‐bowel obstruction due to enterolithiasis that had formed in a duodenal diverticulum, and thus no further cholangiography magnetic resonance imaging (MRI) was performed. Of note, the formation of enteroliths within a duodenal diverticulum is the least common complication of a duodenal diverticulum, and its description is limited to case reports. Obstruction of the small intestine due to the passage of enteroliths from the duodenal diverticulum is equally rare [2]. If conservative treatment fails, fragmentation and milking of enteroliths distal to the colon are the least invasive treatment options. Alternatively, an enterotomy of the stone may be performed, preferably after squeezing it into a nonedematous area [3].

## Ethics Statement

This case report was conducted in accordance with the ethical principles of the Declaration of Helsinki and approved by the Institutional Review Board of First People's Hospital of Huzhou.

## Consent

To protect patient privacy, all data was anonymized, and personal information was handled in strict compliance with relevant regulations and guidelines. The data of this case report will be used solely for academic and research purposes.

## Conflicts of Interest

The authors declare no conflicts of interest.
